# Influenza A Virus Infection Induces Hyperresponsiveness in Human Lung Tissue-Resident and Peripheral Blood NK Cells

**DOI:** 10.3389/fimmu.2019.01116

**Published:** 2019-05-17

**Authors:** Marlena Scharenberg, Sindhu Vangeti, Eliisa Kekäläinen, Per Bergman, Mamdoh Al-Ameri, Niclas Johansson, Klara Sondén, Sara Falck-Jones, Anna Färnert, Hans-Gustaf Ljunggren, Jakob Michaëlsson, Anna Smed-Sörensen, Nicole Marquardt

**Affiliations:** ^1^Department of Medicine Huddinge, Center for Infectious Medicine, Karolinska Institutet, Karolinska University Hospital, Stockholm, Sweden; ^2^Immunology and Allergy, Department of Medicine Solna, Karolinska Institutet, Karolinska University Hospital, Stockholm, Sweden; ^3^Immunobiology Research Program & Department of Bacteriology and Immunology, University of Helsinki, Helsinki, Finland; ^4^HUSLAB, Division of Clinical Microbiology, Helsinki University Hospital, Helsinki, Finland; ^5^Thoracic Surgery, Department of Molecular Medicine and Surgery, Karolinska Institutet, Karolinska University Hospital, Stockholm, Sweden; ^6^Department of Infectious Diseases, Karolinska University Hospital, Stockholm, Sweden; ^7^Division of Infectious Diseases, Department of Medicine Solna, Karolinska Institutet, Stockholm, Sweden

**Keywords:** human, lung, NK cells, influenza A virus, viral pathogenesis, respiratory infections

## Abstract

NK cells in the human lung respond to influenza A virus- (IAV-) infected target cells. However, the detailed functional capacity of human lung and peripheral blood NK cells remains to be determined in IAV and other respiratory viral infections. Here, we investigated the effects of IAV infection on human lung and peripheral blood NK cells *in vitro* and *ex vivo* following clinical infection. IAV infection of lung- and peripheral blood-derived mononuclear cells *in vitro* induced NK cell hyperresponsiveness to K562 target cells, including increased degranulation and cytokine production particularly in the CD56^bright^CD16^−^ subset of NK cells. Furthermore, lung CD16^−^ NK cells showed increased IAV-mediated but target cell-independent activation compared to CD16^+^ lung NK cells or total NK cells in peripheral blood. IAV infection rendered peripheral blood NK cells responsive toward the normally NK cell-resistant lung epithelial cell line A549, indicating that NK cell activation during IAV infection could contribute to killing of surrounding non-infected epithelial cells. *In vivo*, peripheral blood CD56^dim^CD16^+^ and CD56^bright^CD16^−^ NK cells were primed during acute IAV infection, and a small subset of CD16^−^CD49a^+^CXCR3^+^ NK cells could be identified, with CD49a and CXCR3 potentially promoting homing to and tissue-retention in the lung during acute infection. Together, we show that IAV respiratory viral infections prime otherwise hyporesponsive lung NK cells, indicating that both CD16^+^ and CD16^−^ NK cells including CD16^−^CD49a^+^ tissue-resident NK cells could contribute to host immunity but possibly also tissue damage in clinical IAV infection.

## Introduction

Globally, acute respiratory infections cause 4 million deaths every year (1). Of these, infection with seasonal influenza and other respiratory viruses such as respiratory syncytial virus (RSV) constitute a major clinical and economic burden. In the case of influenza virus infections only, 3 to 5 million cases worldwide lead to hospitalization and 300,000 to 650,000 deaths annually^1^. Apart from the general respiratory symptoms, a major complication during different stages of respiratory viral infection is the severe tissue damage affecting the lung. The latter is caused not only by the viral infection and replication in target cells *per se*, but also by influx of and immunological reactions by immune cells including lymphocytes in the lung affecting non-infected cells.

Influenza A virus (IAV)-infected cells have recently been shown to be sensitive to natural killer (NK) cell-mediated cytotoxicity (2), indicating a role of NK cells in the lung upon IAV infection. However, little is known about how IAV infection affects human lung tissue-resident NK (trNK) cells and/or peripheral blood lung-infiltrating NK cells. While human NK cells are largely hyporesponsive in non-infected lungs (3), *in vitro* IAV infection of susceptible cells could readily induce lung NK cell degranulation and IFN-γ production (2, 4). Additionally, studies in murine models have shown that NK cells accumulate in the lung upon IAV infection, contributing to viral clearance (5, 6) and to shaping antiviral responses of cytotoxic T lymphocytes (7). In addition to changes in the lymphocyte composition in the lung, IAV also affects NK cells in other compartments such as the liver. For example, an influenza-specific adaptive-like NK cell subset has been shown to be present in mouse liver but not the lung following infection (8). Both in mice and in humans, a hallmark of hepatic adaptive-like NK cells is high expression of CD49a (9, 10), which is also a hallmark for trNK cells in diverse compartments including the human lung (2, 11) (Marquardt et al., unpublished observations).

IAV-mediated alteration of lung NK cell function and composition might be crucial for disease outcome. Moderate NK cell responses can be beneficial for restricting viral replication (6). However, lung tissue damage mediated by cytotoxic lymphocytes is a frequent complication during infection with RSV (12). Overproduction of NK cell-derived cytokines such as IFN-γ and TNF contributes to severe inflammation during IAV infection (13). It still, however, remains largely unknown how infection with IAV, as well as other respiratory viruses, affects human lung circulating and trNK cells.

Here, we performed a comprehensive assessment of the responsiveness of discrete NK cell subsets from human lung tissue and peripheral blood during *in vitro* and *in vivo* IAV infection. We show that, in particular, CD16^−^ lung and peripheral blood NK cells are strongly primed following viral infection of lung cells. Activated lung trNK cells and NK cells which (re-)circulate to the infected lung likely contribute to host defense but may also exert significant tissue damage. A better understanding of how respiratory viral infections shape NK cell phenotype and function will help in improving and developing new therapeutic approaches for lung-specific pathologies including those caused by respiratory viruses.

## Materials and Methods

### Lung Tissue Collection and Influenza Patients

Clinical samples from seven patients undergoing lobectomy for suspected lung cancer were obtained for this study. None of the patients had received preoperative chemotherapy and/or radiotherapy. Patients had not undergone strong immunosuppressive medication and/or had any hematological malignancy. Clinical and demographic details of patients donating lung tissue are summarized in Table 1. The lung tissue was processed as described before (3).

**Table 1:** Clinical and demographic details of the lung cancer patients included in the study.

**Patient**	**Diagnosis (TNM score)**	**Other comorbidities**	**Medication**	**Smoking (current/ex-smoker/never smoked)**	**Lung function (FEV1% predicted, normal >70)**	**Peripheral blood leukocyte count (normal 4–9 10^**9**^/l)**
59-yo female	Pulmonary adenocarcinoma (ALK positive, T1bN1M0)		None	Ex-smoker	107	4.1
83-yo female	Pulmonary adenocarcinoma (T2bN0M0)	Atrial fibrillation, atherosclerosis, breast cancer (6 yrs ago)	Furosemide, potassium chloride, ezetimibe, omeprazole, spironolactone, warfarin	Occasionally	106	8.8
71-yo female	Pulmonary adenocarcinoma (T1bN0M0)	Atherosclerosis	Acetylsalicylic acid, bisoprolol, felodipine, candesartane, simvastatin, escitalopram	Ex-smoker	93	6.4
69-yo female	Pulmonary adenocarcinoma (T2aN0M0)	Vulvar SQUAMOUS CELL CARCINOMA	None	Ex-smoker	111	5.6
77-yo female	Pulmonary adenocarcinoma (T3N0M0, two tumors in the same lobe, other minimally invasive)	Hypertension, asthma, minimal change nephropathy	Inhaled budesonide, occasional systemic low-dose corticosteroid	Ex-smoker	89	9.3
76-yo male	Squamous cell carcinoma and pulmonary adenocarcinoma (T1aN0M0)	diabetes mellitus type 2, hypertension	Sitagliptin/metformin, acetylsalicylic acid/dipyridamole, amiloride, felodipine/metoprolole, candesartane, simvastatin, finesterid, citalopram	Current smoker	94	7.4
53-yo female	Pulmonary adenocarcinoma (T1aN0M0)	Asthma	Tibolone, desloratadine	Ex-smoker	77	9.6

Peripheral blood was collected from 12 patients with confirmed acute respiratory virus infection during acute phase of infection (1–8 days from onset of symptoms). Samples were also obtained from three patients (non-paired) during convalescence (around 4 weeks after acute virus infection). Of the acutely infected patients, two were receiving bronchial medications at the time of sampling (terbutaline, salbutamol: Short-acting bronchodilators; fluticasone-salmeterol combination therapy: Combination of inhaled cortisone and long-acting bronchodilator). Peripheral blood from 10 age-matched healthy controls was also collected.

The regional review board in Stockholm has approved all studies (lung tissue collection, peripheral blood collection from virus-infected patients and controls), and all donors had given informed written consent prior to collection of samples.

### *In vitro* Infection of Cells With IAV

The influenza A/X31 strain (H3N2 laboratory-adapted strain) was propagated as described before (14). Total mononuclear cells were infected in RPMI1640 medium (Thermo Scientific), supplemented with 10% FCS (Thermo Scientific), 1 mM L-Glutamine (Invitrogen), 100 U/ml penicillin, and 50 μg/ml streptomycin (R10 medium) for 1 h with 5x10^5^ infectious particles of IAV per 10^6^ cells (MOI 0.5), based on TCID50 studies with MDCK cells. Following the infection, cells were washed twice in complete R10 medium and rested or stimulated *in vitro* as described below.

### Functional Analysis of NK Cells

IAV-infected or control mononuclear cells were either rested over night and subsequently cultured in the absence or presence of K562 or A549 target cells (E:T ratio 10:1 and 50:1, respectively) for 6 h, or stimulated with IL-12 (10 ng/ml) and IL-18 (100 ng/ml) for 24 h. During target cell stimulation, anti-CD107a (BV421, H4A3, BD Biosciences) was present throughout the stimulation period, and monensin (GolgiPlug, BD Biosciences) was added during the last 5 h of incubation. For cytokine stimulation, monensin and brefeldin A (GolgiPlug/Stop, BD Biosciences) were added during the last 6 h of incubation.

### Flow Cytometry

Antibodies and clones against the following proteins were used: CD3 (UCHT1, PE-Cy5, Beckman Coulter), CD14 (MϕP9, Horizon V500, BD Biosciences), CD16 (3G8, Brilliant Violet 711 or Brilliant Violet 785, Biolegend), CD19 (HIB19, Horizon V500, BD Biosciences), CD38 (HIT2, Brilliant Violet 711, BD Biosciences), CD45 (HI30, Alexa Fluor 700, Biolegend), CD49a (TS2A, AlexaFluor 647, Biolegend), CD56 (N901, ECD, Beckman Coulter, or HCD56, Brilliant Violet 711, Biolegend), NKG2A (Z1991.10, APC-A780, Beckman Coulter), CD69 (TP1.55.3, ECD, Beckman Coulter), CXCR3 (G025H7, PE-Cy7, Biolegend). After washing twice, cells were stained with streptavidin Qdot 605 or Qdot 585 (both Invitrogen) and Live/Dead Aqua (Molecular probes, Life Technologies). After surface staining, PBMC were fixed and permeabilized using FoxP3/Transcription Factor staining kit (eBioscience). For intracellular staining, the following antibodies were used: granzyme B (GB-11, PE-CF594, BD Biosciences), IFN-γ-Brilliant Violet 570 (4S.B3, Brilliant Violet 570, Biolegend), Ki67 (B56, A700, BD Biosciences), perforin (dG9, PE, Biolegend), and TNF (MAb11, Brilliant Violet 421, Biolegend). IAV infection was monitored using anti-influenza A nucleoprotein-1 (431, Abcam). Samples were analyzed on a BD LSRFortessa equipped with 5 lasers (BD Biosciences), and data were analyzed using FlowJo versions 9.5.2 and 10.5.3 (Tree Star Inc).

### Statistics

GraphPad Prism 6 (GraphPad Software) and SPICE software, version 5.35 (NIAID, NIH), were used for statistical analysis. Wilcoxon matched-pairs signed rank test was used for comparison of matched pairs of data or Mann-Whitney test for comparison of unmatched pairs.

## Results

### IAV Infection Renders Human Lung NK Cells Hyperresponsive

**Figure 1:**
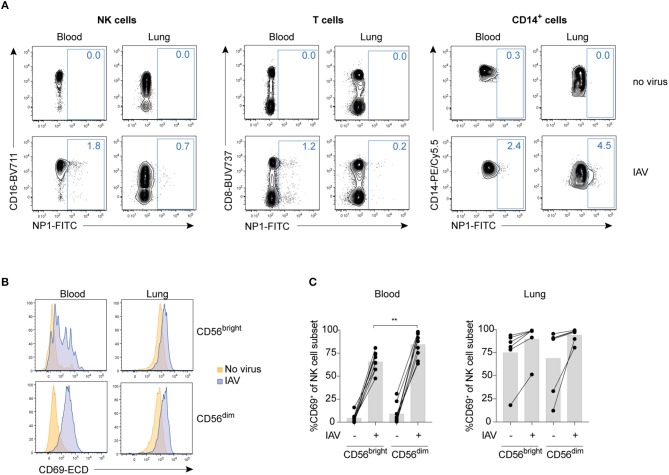
IAV infection of total mononuclear cells activates blood and lung NK cells without infecting them. **(A)** Representative dot plots of NP1 staining in IAV-infected or non-infected blood or lung NK cells (NKG2A^+^ OR CD16^+^ out of live CD3^−^CD14^−^CD19^−^CD45^+^CD56^+^ lymphocytes; left panel), T cells (live CD3^+^CD14^−^CD19^−^CD45^+^ lymphocytes; central panel), or CD14^+^ cells (live CD3^−^CD14^+^CD19^−^CD45^+^ cells; right panel). **(B)** Representative histograms displaying CD69 expression in non-infected (orange) or IAV-infected (blue) CD56^bright^ (top) and CD56^dim^ (bottom) NK cells from blood (left) and lung (right). **(C)** Summary of data displaying CD69 expression on CD56^bright^CD16^−^ (“CD56^bright^”) and CD56^dim^CD16^+^ (“CD56^dim^”) NK cells in blood (left) and lung (right). *n* = 9 (blood), *n* = 7 (lung). Mann Whitney, **p < 0.01.

Compared to peripheral blood NK cells, human lung NK cells are hyporesponsive to target cell stimulation (3). To test whether IAV infection could alter lung NK cell functionality including responsiveness to target cells, we infected mononuclear cells from paired human lung and peripheral blood with the influenza A strain X31 *in vitro*. IAV nucleoprotein 1 (NP1) was readily detectable in particular in lung CD14^+^ cells but also, with lower expression, in NK cells and T cells in blood and lung after 24 h of infection at a MOI of 0.5, indicating that subsets of these cell populations were productively infected (Figure 1A). Despite the lack of productive infection in the vast majority of NK cells, *in vitro* infection with IAV induced a marked increase in CD69 expression on CD56^bright^ and CD56^dim^ NK cells in both blood and lung (Figures 1B,C), indicating that IAV infection of mononuclear cells activates NK cells. Notably, while CD69 indicates early activation of NK cells in peripheral blood (15), it is a hallmark marker for tissue-resident NK cells in diverse tissues including the lung (3). At baseline, ~10% of CD56^dim^ and 80% of CD56^bright^ NK cells in the human lung express CD69 (3). Interestingly, following 24 hours of *in vitro* cell culture, ~80% of CD56^dim^ lung NK cells in most donors expressed CD69 even in the absence of IAV (Figures 1B,C), indicating that long-term cell culture is sufficient to unspecifically induce CD69 on non-tissue-resident lung NK cells.

**Figure 2:**
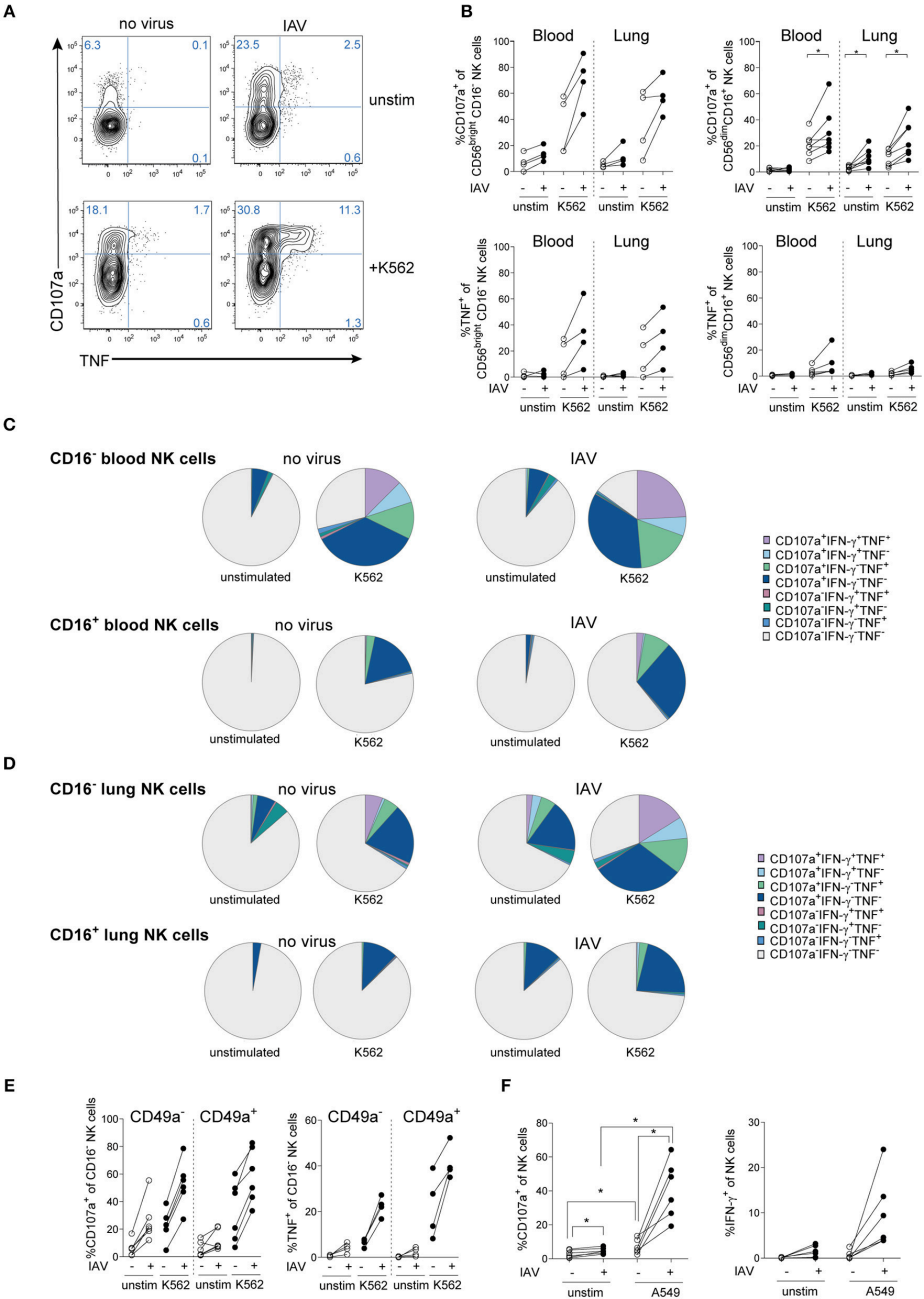
IAV infection induces NK cell hyperresponsiveness in human lung and blood. **(A)** Representative dot plots and **(B)** summary of data displaying degranulation (CD107a expression) and TNF production of total lung NK cells or lung NK cell subsets following IAV infection of lung mononuclear cells and co-incubation with or without K562 target cells (*n* = 4 to 7), *p < 0.05. **(C,D)** SPICE analysis of CD16^−^ (upper panel) and CD16^+^ (lower panel) NK cell responsiveness in **(C)** peripheral blood and **(D)** lung following infection with IAV and co-incubation with or without K562 target cells (*n* = 4). **(E)** Expression of CD107a (left) and TNF (right) in CD49a^−^ and CD49a^+^ CD16^−^ lung NK cells following infection with IAV and co-incubation with or without K562 target cells (*n* = 4 to 7). **(F)** Expression of CD107a and IFN-γ in peripheral blood NK cells following *in vitro* IAV infection of PBMC and co-incubation with or without uninfected A549 cells (*n* = 6), *p < 0.05.

IAV infection also functionally activated human lung NK cells, as evidenced by increased sensitivity to target cells and cytokines (Figure 2A and Figure S1A). In both peripheral blood and lung, IAV induced strong NK cell responses with respect to degranulation following target cell stimulation in both CD56^bright^CD16^−^ and CD56^dim^CD16^+^ NK cells (Figure 2B). Notably, degranulation was if anything stronger in the CD56^bright^CD16^−^ NK cell subset than in the CD56^dim^CD16^+^ subset. IAV infection also induced degranulation even without any additional target cell stimulation in lung and peripheral blood NK cells, but only low levels of cytokine production (Figures 2A–D). IAV-mediated induction of cytokines including TNF and IFN-γ, however, could be boosted by additional stimulation such as with K562 (Figures 2A–D) or IL-12 and IL-18 (Figure S1), respectively.

In addition to CD56^bright^CD16^−^ and CD56^dim^CD16^+^ NK cells, CD56^dim^CD16^−^ NK cells can represent a substantial population in the human lung (2). In order to determine the full spectrum of NK cell responsiveness, we further compared NK cell functions in CD16^−^ versus CD16^+^ NK cells in lung and blood (Figures 2C,D). In comparison to CD56^bright^CD16^−^ NK cells (Figures 2A,B), total CD16^−^ NK cells displayed a stronger CD107a^+^ response in blood and lung (Figures 2C,D), indicating that CD56^dim^CD16^−^ NK cells contribute to the NK cell response.

Furthermore, the response to IAV infection alone without additional stimulus in CD16^−^ lung NK cells was similar to that of CD16^−^ lung NK cells stimulated with K562 cells in the absence of IAV infection (Figure 2D), thus showing that IAV-infection alone can drive a strong NK cell response. From a qualitative point of view, IAV infection induced a marked polyfunctional response in CD16^−^ NK cells in the lung, both with and without additional stimulation by K562 cells (Figure 2D). In contrast, IAV infection mainly induced a stronger degranulation in CD16^+^ NK cells, with less induction of TNF and IFN-γ compared to CD16^−^ NK cells (Figure 2D). Thus, IAV infection affects mainly CD16^−^ NK cells in human lung and peripheral blood. In the human lung, CD16^−^ NK cells are partly constituted by trNK cells as identified by expression of CD69, CD49a, and/or CD103 (2, 3). Due to their location, trNK cells are likely to be directly exposed to virus-infected neighboring tissue cells including epithelial cells and alveolar macrophages upon respiratory virus infection *in vivo*. Within the CD16^−^ NK cell subset, CD49a^+^ trNK cells were functionally competent and were strongly primed by IAV infection *in vitro*, with a trend toward increased TNF production by CD49a^+^ NK cells as compared to CD49a^−^ NK cells (Figure 2E). Activation of discrete NK cell subsets in lung and peripheral blood by respiratory virus infection might have physiological consequences upon infection, as indicated by RSV infection in mice (12). In line with that, IAV-primed peripheral blood NK cells became strongly sensitive to A549 cells, a lung epithelial cell line which is normally not lysed by resting NK cells (Figure 2F). This suggest a potential contribution of blood NK cells to host responses but potentially also to lung tissue-damage during IAV infection, since circulating NK cells constitute the vast majority of NK cells in the human lung (3).

### IAV Infection Activates Human Peripheral Blood NK Cell Subsets *in vivo*

**Figure 3:**
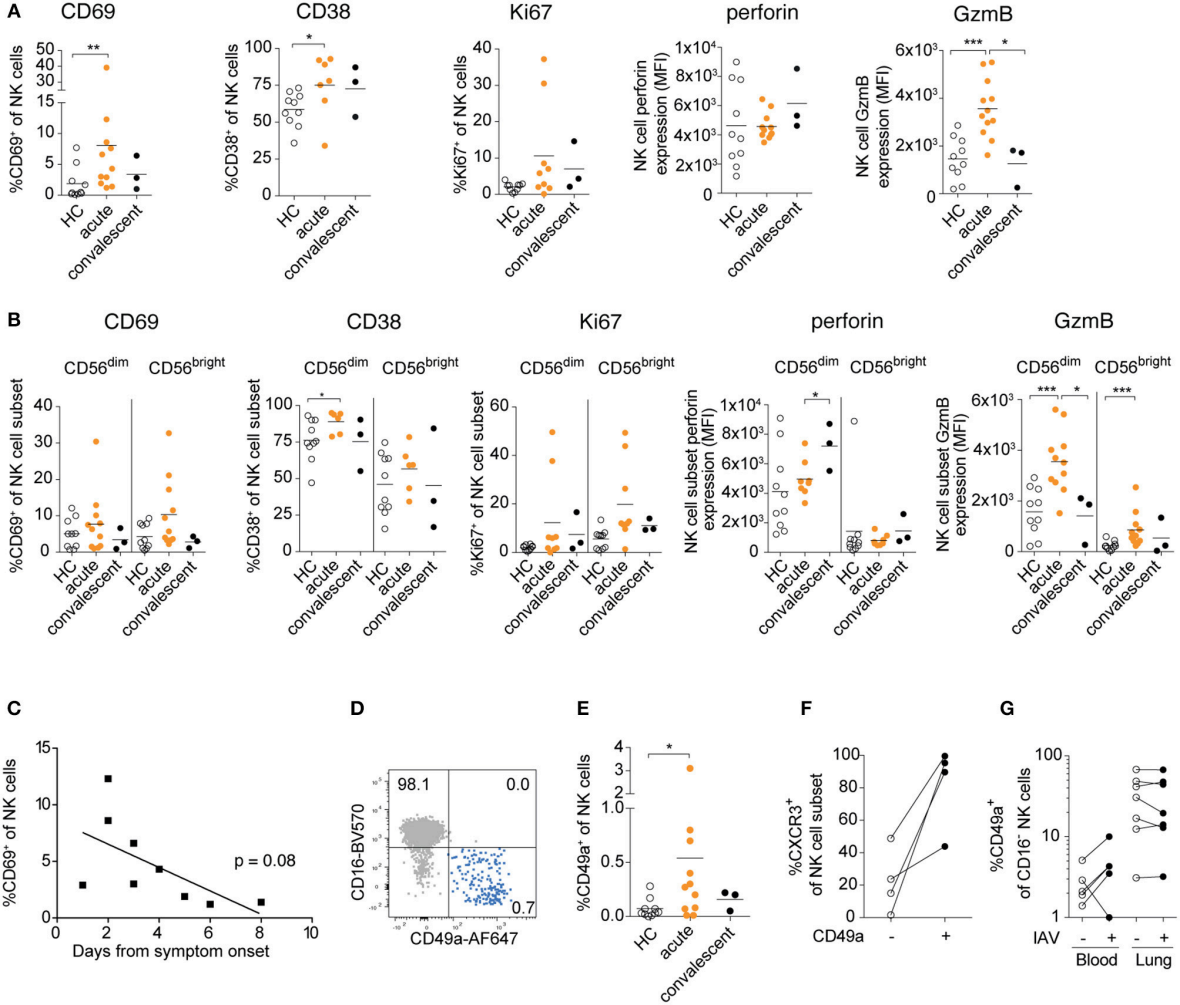
Human peripheral blood NK cells are activated and upregulate CD49a upon acute IAV infection. **(A)** Frequencies of CD38^+^, CD69^+^, Ki67^+^, and CXCR3^+^ NK cells and expression levels of granzyme B and perforin in NK cells in age- and sex-matched healthy controls (HC). Patients were studied during the acute and convalescent phase of IAV infection. **(B)** Summary of data of CD38, CD69, Ki67, and granzyme B expression in CD56^dim^CD16^+^ and CD56^bright^CD16^−^ NK cells during acute and convalescence phase of IAV infection as compared to age- and sex-matched healthy controls. **(A,B)** Mann-Whitney, *n* = 10 (HC), *n* = 6 to 12 (IAV acute), *n* = 3 (IAV convalescent), *p < 0.05, **p < 0.01, ***p < 0.001. **(C)** Frequency of CD69^+^ NK cells during acute phase of IAV infection in relation to the days past from symptom onset, *n* = 9. **(D)** Representative dot plot displaying CD16 and CD49a expression on total peripheral blood NK cells during acute IAV infection. **(E)** *Ex vivo* expression of CD49a on NK cells from patients during acute and convalescent phase of IAV infection and healthy controls. Mann-Whitney, *n* = 10 (HC), *n* = 11 (IAV acute), *n* = 3 (IAV convalescent), *p < 0.05. **(F)** *Ex vivo* expression of CXCR3 on CD49a^−^ and CD49a^+^ blood NK cells during acute IAV infection, *n* = 4. **(G)** Expression of CD49a on CD16-blood and lung NK cells following *in vitro* IAV infection, *n* = 5 (blood), *n* = 7 (lung).

In order to determine the extent to which IAV infection promotes activation of circulating NK cells *in vivo*, we assessed the activation of peripheral blood NK cells in patients with confirmed IAV infection during different stages of the disease (Figure 3). During the acute phase of the infection, increased expression of CD69, CD38, Ki67, and granzyme B was observed on NK cells indicating activation (Figure 3A).

While both CD56^dim^ and CD56^bright^ NK cells displayed signs of activation, the two subsets differed in their responses: Expression of CD69 and Ki67 were particularly elevated in CD56^bright^ NK cells, whereas granzyme B expression was higher in CD56^dim^ NK cells (Figure 3B). Interestingly, the expression levels of perforin increased in the CD56^dim^ NK cells even in the convalescent phase, in contrast to all other activation markers analyzed, indicating potential long-term imprinting by respiratory virus infection (Figure 3B). Together, our data reveal that IAV infection *in vivo* activates NK cells in the acute stages of infection, with distinct responses in CD56^bright^ and CD56^dim^ NK cells.

As described above, IAV infection activated peripheral blood NK cells *in vivo* as indicated by CD69 expression, and we detected a trend toward increased activation levels at the earliest stages of acute infection (2–4 days after symptom onset) (Figure 3C). It remains to be determined, however, whether human peripheral blood NK cells have been locally activated in the lung tissue or whether they have been activated systemically. Notably, a small but detectable population of CD16^−^CD49a^+^ NK cells was identified in the blood during the acute phase of infection, but not in the convalescent phase or in healthy donors (Figures 3D,E). These CD49a^+^ NK cells strongly co-expressed the lung-homing receptor CXCR3 (Figure 3F). Induction of CD49a on blood NK cells could also be observed upon *in vitro* IAV infection, whereas CD49a expression was not further increased on lung NK cells (Figure 3G). Elevated expression of CD69 and CD49a might facilitate the infiltration of NK cells into the lung tissue upon infection, suggesting that circulating activated NK cells could extend the pool of trNK cells in the human lung, further contributing to host immune responses but potentially also to tissue damage.

## Discussion

NK cells are the most frequent innate lymphoid cells in human lung tissue (3), and the vast majority of NK cells in the human lung are circulating between the organ and perhipheral blood (3). Respiratory virus infections such as IAV and RSV are likely to affect not only NK cells residing in the lung but also NK cells in the circulation or cells circulating between the lung and peripheral blood. Futhermore, both circulating and lung trNK cells likely contribute considerably not only to host protection but also to possible adverse effects in the tissue during respiratory infection. Here, we characterized the NK cell response to IAV infection in human lung and blood, both *in vitro* and *ex vivo*, and demonstrate marked viral infection-induced hyperresponsiveness, particularly in CD56^bright^CD16^−^ NK cells.

While confirming that IAV infection is able to override lung NK cell hyporesponsiveness (2), Cooper et *al*. found no functional differences between CD56^dim^ and CD56^bright^ NK cells following IAV infection in lung tissue explants. The difference between their results and the present results might possibly be due to differences in infection and cell activation in cell suspensions vs. tissue explants. In the tissue explants, limited exposure of lung NK cells to IAV and activated accessory cells and/or co-localization with alveolar macrophages might result in lower NK cell activation potential. While usage of tissue explants might be more physiological due to maintaining the tissue structure and lymphocyte localization in the tissue, *in vitro* infection in single cell suspensions allows investigations into the overall response capacity of the total NK cell population present in the tissue. Adding to these results, our findings demonstrate that IAV infection not only activates human lung NK cells but also peripheral blood NK cells, which are recruited in high numbers to the lung upon viral infection (5, 6) and are likely to considerably contribute to lung tissue damage.

In acutely infected IAV patients, serum/plasma levels of IL-6, IL-8, IL-15, and CXCL10 (IP-10) are elevated (13, 16, 17), and airway epithelial cells and alveolar macrophages release CXCL10 and IL-15 in the lung upon stimulation with IFN-γ, viral infection, or other pulmonary diseases (18–22). Together, this altered cytokine mileu in infected lung tissue is likely to induce priming specifically of CD56^bright^CD16^−^ NK cells. In addition, direct cell contact with infected target cells can contribute to subset-specific NK cell activation (2, 23). However, IAV infection increased peripheral blood NK cell responsiveness also toward non-infected lung epithelial cells which are normally NK cell resistant, indicating that healthy non-infected lung epithelial cells can be targeted by human NK cells upon respiratory viral infection, consistent with studies in mice (12).

In the lung tissue, a subset of CD56^bright^CD16^−^ NK cells can be identified as trNK cells, co-expressing CD69, CD49a, and/or CD103 (2, 3). *In vitro* IAV infection strongly primed CD16^−^CD49a^+^ trNK cells toward target cell-responsiveness, indicating that not only activated circulating NK cells entering the lung but also trNK cells get activated and, hence, might contribute to host response and potential tissue damage. Furthermore, despite overall low expression, CD49a was upregulated *in vivo* on a subset of blood NK cells during the acute phase of IAV infection. These CD49a^+^ NK cells strongly co-expressed CXCR3, which might facilitate specific recruitment of this subset to the lung. Several studies have demonstrated elevated levels of CXCR3 ligands in the context of airway inflammation in chronic obstructive pulmonary disease (24), asthma (25, 26) and chronic lung allograft dysfunction (27, 28). Moreover, elevated levels of CXCL10 are present in the circulation during acute IAV infection (16) and are linked to pathogenicity of severe IAV infection (29). In mice, recruitment of NK cells to the lung upon IAV infection was shown to be partially dependent on expression of CXCR3 (30). Additionally, IAV-activated blood NK cells might also be recruited to other compartments than the lung, similar to recruitment of lung-specific T cells to the small intestine upon respiratory influenza infection in mice, causing tissue injury and intestinal disease (31). Thus, elevated expression of receptors promoting tissue-invasion of peripheral blood NK cells might contribute to host response and possible disease-associated pathology. Lastly, elevated levels of CD49a on CD16^−^ blood but not lung NK cells suggests either egress of activated trNK cells from the lung into the circulation upon acute infection, or cytokine-induced upregulation of CD49a expression in blood, lung, or other compartments (32).

**Figure 4:**
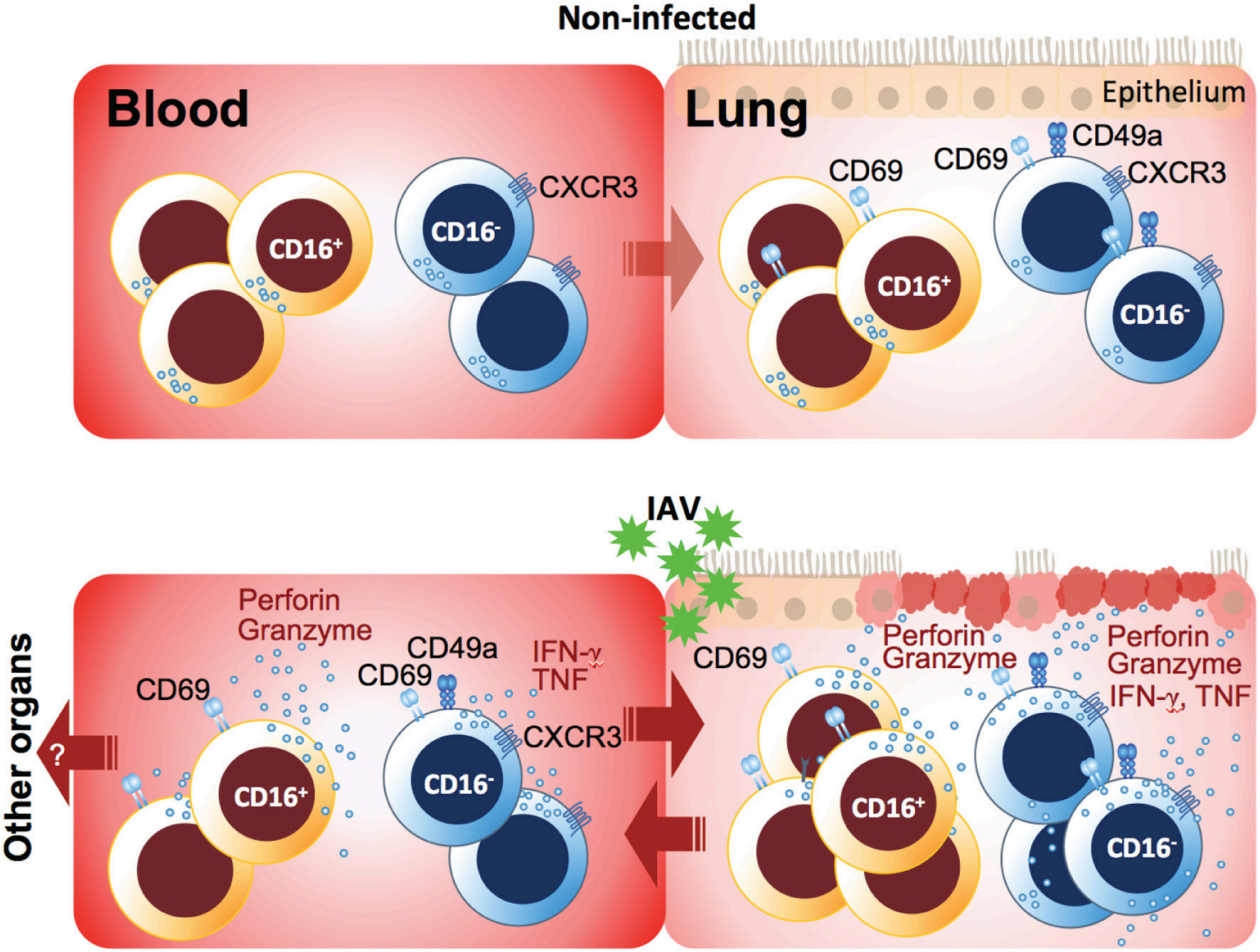
Model of peripheral blood and lung NK cell activation in acute respiratory IAV infection. While NK cells in non-infected lung are hyporesponsive, IAV infection induced rapid upregulation of CD69 and unspecific degranulation in particular, but not exclusively, in CD16^−^ NK cells both in blood and lung during the acute phase of infection. The presence of target cells or cytokines boosted these NK cell responses and, in addition, induced cytokine production. In peripheral blood, CD16^+^ NK cells displayed mainly cyotoxic characteristics including increased levels of effector molecules, whereas a small subset of CD16^−^ NK cells co-expressing the integrin CD49a and the lung-homing chemokine receptor CXCR3 emerged during the acute phase of infection. CD49a and CXCR3 likely facilitate tissue-homing and establishment of tissue-residency in the IAV-infected lung but potentially also in other organs. Finally, functionally primed NK cells accumulate in the lung, and both recruited peripheral blood NK cells but also tissue-resident NK cells may contribute to tissue damage through unspecific and/or specific lysis of lung epithelial cells.

Together, our data demonstrate that NK cells in blood and lung are tightly regulated and linked to each other upon respiratory viral infection (Figure 4). IAV infection-mediated hyperresponsiveness of peripheral blood and lung NK cells might considerably contribute to tissue injury in the lung, promoted by increased tissue-homing capacity upon infection. Identifying and targeting these hyperresponsive NK cells in viral infections might be a future therapeutic approach for reducing respiratory virus-induced tissue pathologies.

## Ethics Statement

This study was carried out in accordance with the recommendations of the regional ethical review board in Stockholm with written informed consent from all subjects. All subjects gave written informed consent in accordance with the Declaration of Helsinki. The protocol was approved by the regional ethical review board in Stockholm.

## Author Contributions

MS, JM, and NM: conceptualization. SV, JM, and NM: methodology. MS, EK, and NM: investigation. NJ, KS, SF-J, AF, AS-S, MA-A, and PB: resources. MS and NM: writing—original draft. MS, JM, NM, and H-GL: writing—review and editing. NM: visualization. AS-S, JM, NM, and H-GL: funding acquisition.

### Conflict of Interest Statement

The authors declare that the research was conducted in the absence of any commercial or financial relationships that could be construed as a potential conflict of interest.

## Footnotes

^1^http://www.who.int/news-room/fact-sheets/detail/influenza-(seasonal)

## Abbreviations

IAV: Influenza A virus
RSV: Respiratory syncytial virus
trNK cells: tissue-resident NK cells.
