# Supplementary figures and images for: Influenza A Virus Infection Induces Hyperresponsiveness in Human Lung Tissue-Resident and Peripheral Blood NK Cells
Source: Front Immunol. 2019 May 17;10:1116. doi: 10.3389/fimmu.2019.01116 (PMC6534051; doi:10.3389/fimmu.2019.01116)

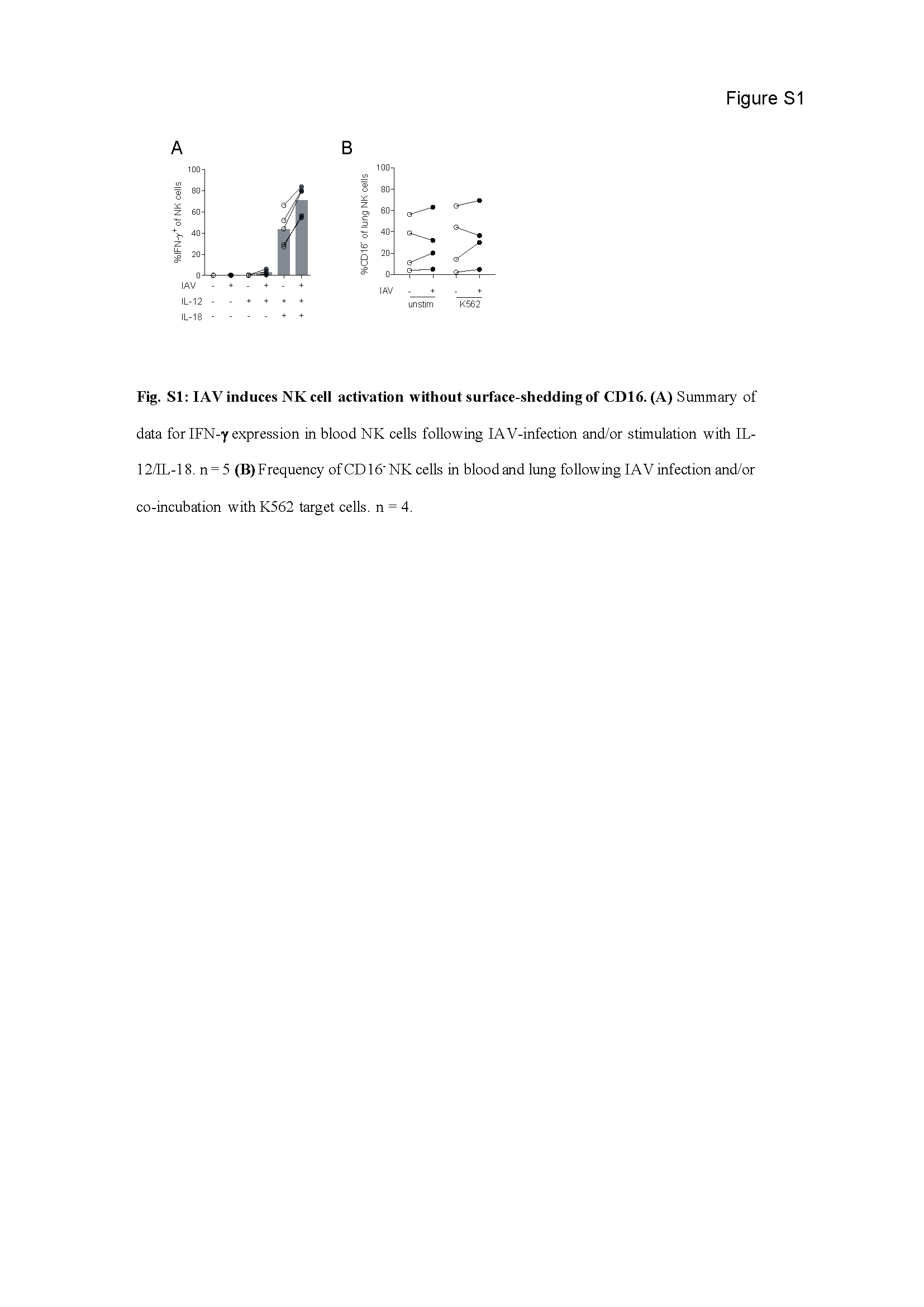

Supplement: Supplementary file 1 [file Image_1.TIFF]
